# Case Report: Laparoscopic management of a primary omental pregnancy concealed beneath the bowel

**DOI:** 10.3389/fmed.2026.1938779

**Published:** 2026-10-07

**Authors:** Jiaqi Shi, Xiaoteng Qiang, Cuihong Ji, Siyi Lian, Xuxing Zhang, Wei Liu

**Affiliations:** 1 Department of Obstetrics and Gynecology, Beijing Shunyi District Maternal and Child Health Hospital (Shunyi Maternal and Children’s Hospital of Beijing Children’s Hospital), Beijing, China; 2 Department of Obstetrics and Gynecology, Fu Xing Hospital, Capital Medical University, Beijing, China

**Keywords:** abdominal pregnancy, case report, ectopic pregnancy, laparoscopy, omental pregnancy

## Abstract

Primary omental pregnancy is an extremely rare form of ectopic pregnancy. We report a case of successful laparoscopic management in a 19-year-old nulliparous woman with no traditional risk factors. Despite sonographic evaluation, including transvaginal, transabdominal, and multi-organ abdominal ultrasonography, the ectopic lesion was not identified preoperatively. Diagnostic laparoscopy was performed, but initial pelvic exploration was also negative. Only after lifting the bowel was a 2 cm omental pregnancy discovered, concealed beneath the bowel, and completely excised. Postoperatively, serum beta-human chorionic gonadotropin (β-hCG) declined by 70.3% on day 1 and 95.3% on day 4 without methotrexate use. This case underscores two points: first, systematic laparoscopic exploration beyond the pelvis is essential when initial inspection is negative; second, the absence of traditional risk factors does not exclude rare-site ectopic implantation.

## Introduction

Ectopic pregnancy accounts for approximately 1%–2% of all pregnancies and is a leading cause of early pregnancy-related maternal mortality (1). Abdominal pregnancy is a rare subtype, accounting for less than 1% of all ectopic pregnancies (2, 3). Omental pregnancy is among the rarest forms, with only 16 cases identified in a review from 1958 to 2012 (4) and 36 cases in a Chinese review from 1978 to 2015 (5). The diagnosis is challenging because omental pregnancy lacks the typical sonographic features of tubal ectopic pregnancy and the lesion is often located far from the pelvis, leading to delayed diagnosis or incidental discovery during emergency surgery (6, 7). Rupture can result in life-threatening intra-abdominal hemorrhage. The reported mortality rate of 2%–30% relates to abdominal pregnancy in general and may not be specific to early omental pregnancy (2). Here we report a case of omental pregnancy in a young nulliparous woman with no traditional risk factors. The clinical and histopathological features favored primary implantation, although secondary implantation could not be entirely excluded; the lesion was concealed beneath the bowel and missed on preoperative imaging. We describe the systematic laparoscopic approach that led to its identification and complete excision.

**Table 1:** Timeline of the case.

Date	Clinical event	Key imaging and laboratory findings	Management and follow-up
2025-04-17	Initial presentation	β-hCG 4,198 mIU/mL; TVUS: empty uterus, atypical left adnexal mass (1.9 × 1.6 cm), pelvic fluid 2.8 × 1.1 cm	Admission, close monitoring
2025-04-18	Repeat evaluation and surgery	β-hCG 4,236 mIU/mL; pelvic fluid increased to 5.7 × 2.3 cm; transabdominal US unremarkable	Laparoscopic exploration; complete excision of omental mass; left ovarian cystectomy
2025-04-19	Postoperative day 1	β-hCG 1,259.33 mIU/mL (70.3% decrease); Hb 113 g/L, Hct 32.9%	Uneventful recovery
2025-04-22	Postoperative day 4	β-hCG 197.68 mIU/mL (95.3% decrease)	Continued recovery
2025-04-23	Postoperative pathology report	Omental specimen: chorionic villi and trophoblastic cells; left ovarian corpus luteum cyst with hemorrhage	Confirmed omental ectopic pregnancy
2025-05-07	Postoperative day 19	β-hCG 2.22 mIU/mL (below upper limit of normal)	Outpatient follow-up
2025-05-13	Postoperative day 25	β-hCG 0.98 mIU/mL	Outpatient follow-up
2025-05-16	Postoperative day 28	Menstruation resumed	-
2025-05-26	Postoperative day 38	β-hCG 0.76 mIU/mL	-
2025-06-19	Last clinical follow-up (8 weeks)	-	No symptoms; emotionally stable; mild pain resolved within 2 days; returned to daily activities within 7 days; satisfied with treatment

The patient remained asymptomatic with stable vital signs throughout the clinical course. Blood pressure was 112/75 mmHg and pulse was 93 beats/min at presentation.

## Case presentation

A 19-year-old woman, gravida 1 para 0 (G1P0), presented with 57 days of amenorrhea and a positive home urine pregnancy test on April 17, 2025. Her menstrual cycles were regular, with bleeding for 5 days every 30 days, moderate flow, and dysmenorrhea (VAS score 8). Her last menstrual period was February 20, 2025. She denied nausea, vomiting, abdominal pain, or vaginal bleeding. She was sexually active with barrier contraception but not consistently. Her past medical history was unremarkable, with no history of surgery or pelvic inflammatory disease. She had a 1-year smoking history of about 5 cigarettes per day, and had quit smoking 2 days before presentation. Family history: her mother and one elder sister both had polycystic ovary syndrome.

Physical examination: temperature 37.1 °C, pulse 93 beats/min, respiration 18 breaths/min, blood pressure 112/75 mmHg. Abdomen was soft and non-tender. Pelvic examination: external genitalia were normal; vagina was patent with a small amount of discharge; cervix was normal size with mild ectropion, no contact bleeding, no cervical motion tenderness; uterus was anteverted, normal size, mobile, non-tender; bilateral adnexa were thickened but non-tender.

Investigations: On April 17, 2025, serum beta-human chorionic gonadotropin (β-hCG) was 4198.00 mIU/mL. Transvaginal ultrasound showed: uterus anteverted, size 4.8 × 4.2 × 3.1 cm, a 1.0 × 0.5 cm hypoechoic nodule in the anterior wall (suspected small fibroid); endometrial thickness 8.8 mm, no gestational sac in the cavity. Left ovary: a 3.7 × 2.6 cm cystic mass with septations and flocculent echoes, no significant flow on color Doppler; adjacent to the left ovary a 1.9 × 1.6 cm heterogeneous hypoechoic area with slightly rich blood flow (RI 0.53). Right ovary: 3.6 × 1.5 cm with ≥12 small anechoic areas (polycystic-like change). The pouch of Douglas contained a fluid collection (described as a liquid anechoic area without further characterization), measuring 2.8 × 1.1 cm. On April 18, 2025, repeat serum β-hCG was 4,236.00 mIU/mL. Repeat ultrasound showed: left ovarian cyst 3.5 × 2.4 cm with persistent septations and flocculent echoes; the heterogeneous area adjacent to the left ovary was unchanged; the pelvic fluid collection had increased to 5.7 × 2.3 cm. Transabdominal ultrasound of the liver, gallbladder, pancreas, spleen, kidneys, and ureters revealed no abnormalities. Preoperative laboratory tests showed the following: white blood cell count 4.74 × 10⁹/L, red blood cell count 4.0 × 10^12^/L, hemoglobin 120 g/L, hematocrit 35.1%, mean corpuscular volume 87.8 fL, mean corpuscular hemoglobin 30 pg, and platelet count 261 × 10⁹/L. Blood group was A, Rh-positive. Renal, liver, and coagulation function tests were normal. Postoperative repeat testing on April 19 showed: white blood cell count 6.1 × 10⁹/L, red blood cell count 3.69 × 10^12^/L, hemoglobin 113 g/L, hematocrit 32.9%, mean corpuscular volume 89.2 fL, mean corpuscular hemoglobin 30.6 pg, and platelet count 235 × 10⁹/L.

Given the elevated β-hCG (4,236 mIU/mL), the empty uterine cavity, and an atypical extrauterine mass not consistent with a typical tubal ectopic pregnancy on ultrasound in consultation with the ultrasonographer, a non-tubal ectopic pregnancy was suspected, and an abdominal pregnancy could not be excluded. After thorough discussion of the alternatives, including continued β-hCG and ultrasound monitoring, uterine aspiration, MRI, and medical management, the patient, who had no fertility plans and considered MRI expensive and time-consuming, preferred prompt surgical management. Laparoscopic exploration was performed under general anesthesia on April 18, 2025. Intraoperative findings and procedure: Laparoscopy revealed approximately 50 mL of hemoperitoneum (Figure 1A). After confirming that both tubes, ovaries, and the pelvis were free of gestational tissue, the exploration was extended systematically to the upper abdomen, including inspection of the diaphragm, liver surface, bowel, mesentery, paracolic gutters, and remaining peritoneal surfaces. A bluish-purple, friable mass of about 2 cm in diameter was identified on the omental surface, initially concealed by overlying bowel loops and exposed by gentle lateral retraction of the bowel (Figure 1B). Bipolar coagulation was applied to precoagulate the omentum approximately 1 cm around the lesion, and the gestational tissue was completely resected along the precoagulated margin, with no bleeding. The left ovary was then explored; it was enlarged to about 5 cm, including the cyst. The cyst wall appeared thin, and a small bleeding point was noted on its surface. The ovarian cortex was incised, the corpus luteum cyst was exposed and completely enucleated, and the ovary was reconstructed with 2-0 absorbable suture. The omental pregnancy specimen and the cyst wall were placed in an endoscopic bag. The abdominopelvic cavity was irrigated with warm saline, and after confirming no active bleeding, the bag was removed through the umbilical port. The CO₂ was evacuated and the incisions were closed. The patient's intraoperative course was uneventful, with intraoperative blood loss of about 10 mL, infusion of 1,000 mL, and urine output of 100 mL.

**Figure 1:**
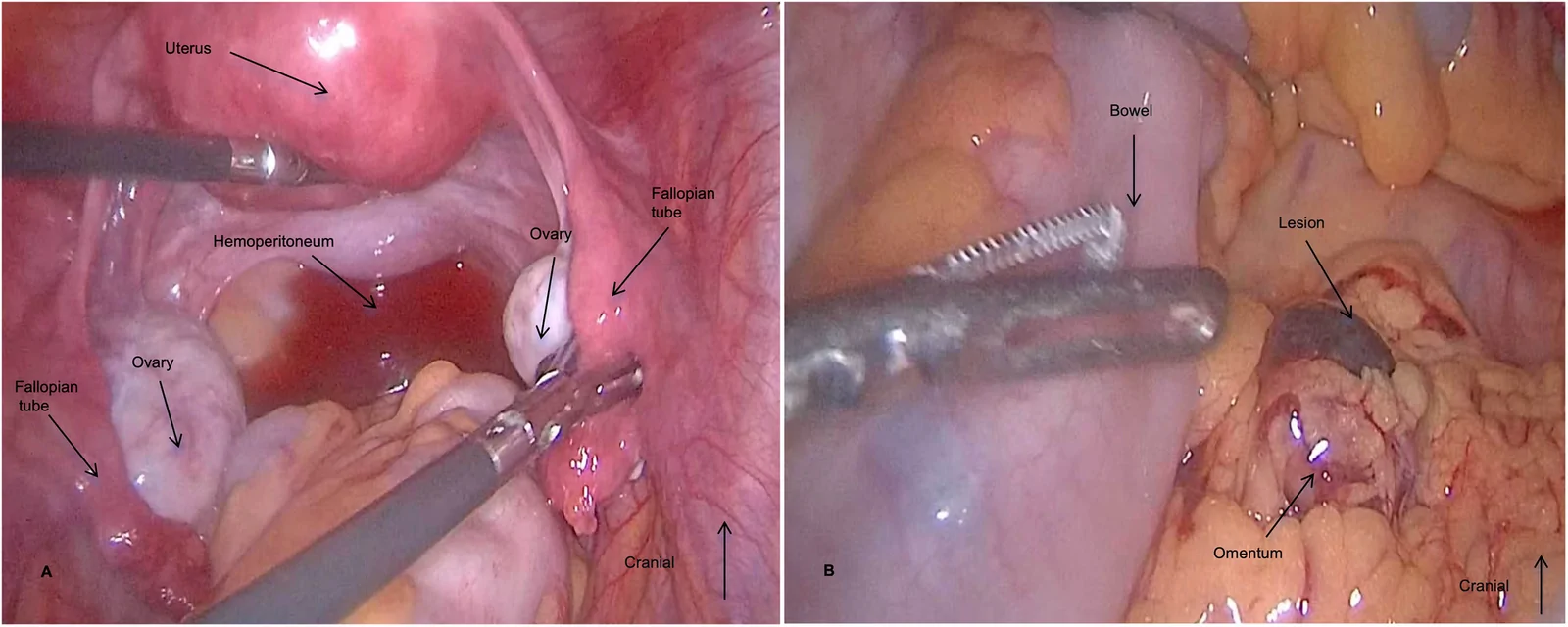
Laparoscopic findings. In both panels, the cranial side is at the top. **(A)** Initial pelvic exploration showing approximately 50 mL of hemoperitoneum; the uterus and bilateral fallopian tubes appeared normal, with no gestational tissue identified. The left ovary contained a corpus luteum cyst. **(B)** After lateral retraction of the bowel, a bluish-purple friable mass approximately 2 cm in diameter is identified on the omental surface (arrow). The surrounding bowel and omentum are labeled.

Postoperative recovery: The patient recovered uneventfully, without abdominal pain or fever. Methotrexate was not given. Serial serum β-hCG measurements: postoperative day 1 (April 19) 1,259.33 mIU/mL (70.3% decrease from preoperative 4,236 mIU/mL); day 4 (April 22) 197.68 mIU/mL (95.3% decrease); day 19 (May 7) 2.22 mIU/mL; day 25 (May 13) 0.98 mIU/mL; day 38 (May 26) 0.76 mIU/mL. Menstruation resumed on May 16 (postoperative day 28) with normal cycle and flow (Figure 2).

**Figure 2:**
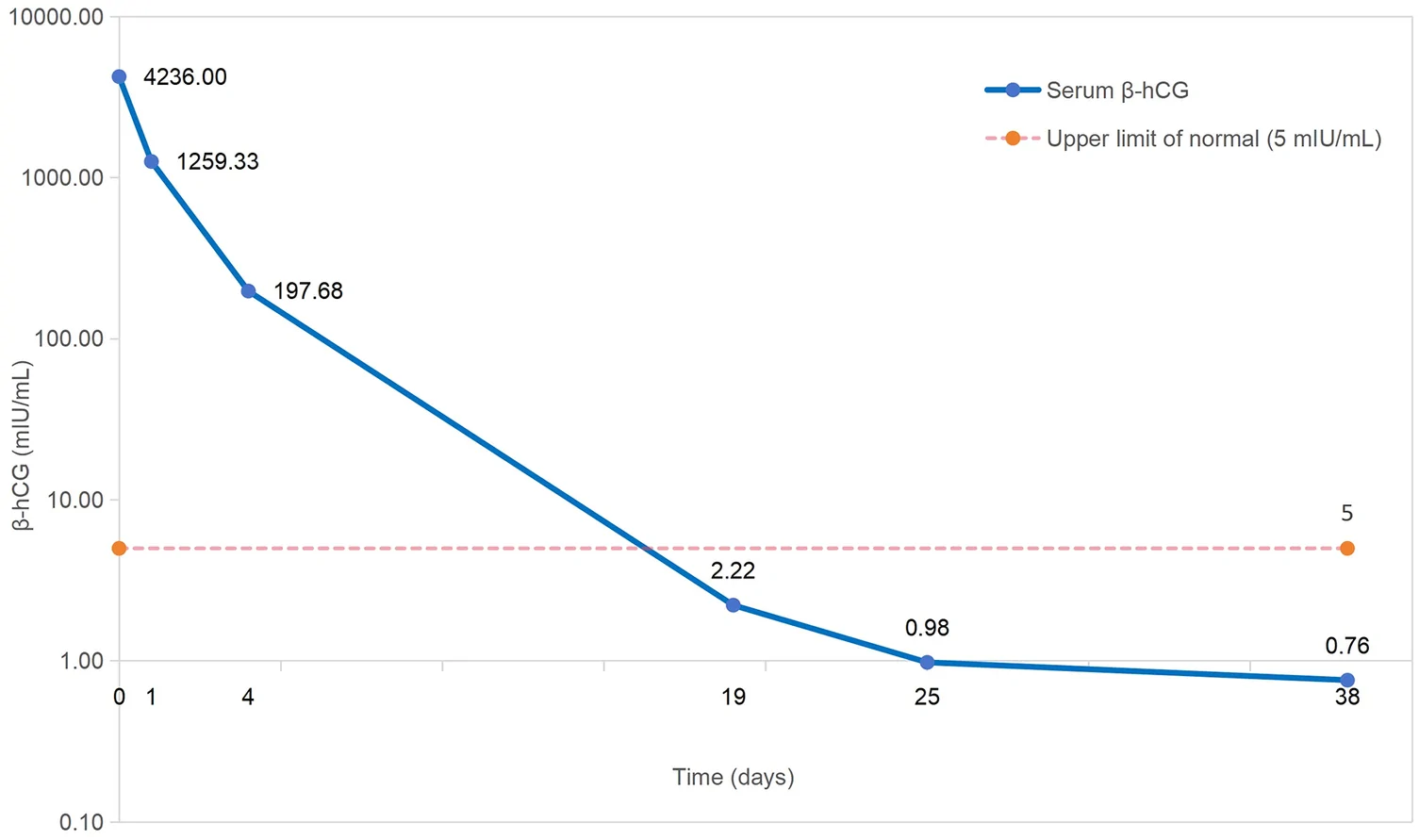
Serum β-hCG trend on a logarithmic scale. Day 0 represents the preoperative baseline (4,236 mIU/mL); days 1, 4, 19, 25, and 38 are postoperative days. Serum β-hCG declined to 1,259.33 mIU/mL on day 1, to 197.68 mIU/mL on day 4, to 2.22 mIU/mL on day 19, to 0.98 mIU/mL on day 25, and to 0.76 mIU/mL on day 38. The dashed line indicates the upper limit of normal (5 mIU/mL).

Description of tissue specimen morphology: Left ovarian cyst specimen: fragmented cystic wall, total size 2.5 × 2.5 × 1 cm, wall thickness 0.1–0.2 cm, gray-white to gray-yellow, partly gray-brown, soft to firm. Omental specimen: irregular tissue, total size 4.5 × 2 × 0.6 cm, gray-yellow to gray-brown, with villus-like structures, soft. Pathology: The omental specimen showed chorionic villi and identifiable trophoblastic cells, consistent with omental ectopic pregnancy (Figure 3A). The resection margins were not assessed, as margin evaluation is not routinely reported for gestational tissue pathology. Microscopic diagnosis: Left ovarian cyst consistent with corpus luteum cyst with hemorrhage and ovarian follicular cyst (Figure 3B).

**Figure 3:**
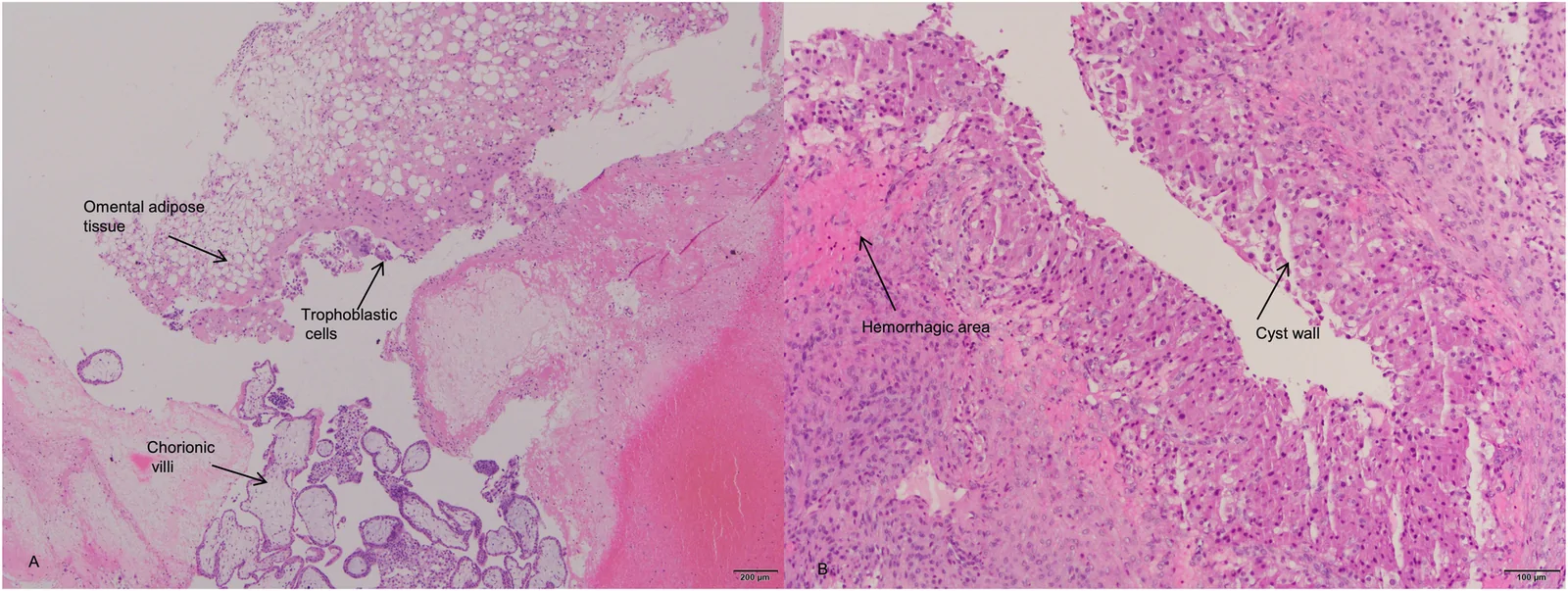
Histopathological examination (hematoxylin and eosin staining). **(A)** Omental specimen (original magnification ×40) showing chorionic villi and trophoblastic cells within omental adipose tissue, consistent with omental ectopic pregnancy. Scale bar: 200 µm. **(B)** Left ovarian corpus luteum cyst (original magnification ×100) showing cyst wall and hemorrhagic areas. Scale bar: 100 µm.

The patient was discharged on postoperative day 3. The last clinical follow-up was on June 19, 2025, which was 8 weeks after surgery. At that visit, she reported no symptoms, and menstruation had returned to normal. There were no postoperative complications, adverse events, readmissions, reinterventions, or treatment intolerance during the follow-up period. The patient reported mild postoperative pain that resolved within 2 days, and she returned to normal daily activities within 7 days. She remained emotionally stable throughout the follow-up period and expressed satisfaction with the treatment at the last visit. A comprehensive timeline of the diagnostic evaluation, surgical management, and postoperative follow-up is provided in (Table 1).

## Discussion

Omental pregnancy is among the rarest forms of abdominal pregnancy. Maiorana et al. identified only 16 reported cases between 1958 and 2012 (4), and a Chinese review from 1978 to 2015 included only 36 cases (5). Most cases are diagnosed during emergency surgery for acute abdomen.

### Establishing the diagnosis of primary omental pregnancy

The diagnosis of primary omental pregnancy requires fulfillment of the accepted criteria for primary peritoneal pregnancy: normal bilateral fallopian tubes and ovaries, absence of a uteroperitoneal fistula, and implantation occurring sufficiently early to exclude secondary implantation (8). In our patient, both fallopian tubes appeared normal at laparoscopy, with no evidence of tubal pregnancy, tubal abortion, or rupture. Both ovaries were free of gestational tissue; the left ovary contained a corpus luteum cyst, which was enucleated. There was no history of uterine perforation, and no uteroperitoneal fistula was identified. Histopathological examination of the omental specimen revealed chorionic villi and identifiable trophoblastic cells, consistent with omental ectopic pregnancy.

We acknowledge that normal-appearing fallopian tubes at laparoscopy cannot entirely exclude a prior small tubal pregnancy that may have aborted spontaneously, with subsequent secondary omental implantation. However, no clinical, sonographic, or intraoperative evidence of tubal pregnancy was found. The absence of an adnexal gestational mass, the normal appearance of both fallopian tubes, and the histopathological finding of trophoblastic tissue within the omentum collectively favor a primary omental pregnancy; however, secondary implantation cannot be entirely excluded.

### Diagnostic challenges and preoperative recognition

The patient was initially classified as having a pregnancy of unknown location. The differential diagnosis included early or failing intrauterine pregnancy, completed miscarriage, tubal ectopic pregnancy, ovarian ectopic pregnancy, hemorrhagic or corpus luteum cyst, and abdominal pregnancy. Transvaginal ultrasound demonstrated an empty uterine cavity with no gestational sac, making a viable intrauterine pregnancy unlikely, although an early failing intrauterine pregnancy could not be entirely excluded. The absence of vaginal bleeding and the elevated β-hCG level argued against a completed miscarriage. A typical tubal ectopic pregnancy was considered, but no adnexal ring sign or separate tubal mass was identified. A left ovarian cystic mass with septations and flocculent echoes was noted, most consistent with a corpus luteum cyst, although an ovarian ectopic pregnancy could not be definitively excluded. The progressive increase in pelvic fluid, together with the absence of an intrauterine or tubal pregnancy, raised concern for an abdominal pregnancy. The serum β-hCG rose from 4,198 to 4,236 mIU/mL over approximately 24 h, an increase of less than 1%. This rise is more suggestive of an ectopic pregnancy than of a normal intrauterine pregnancy. Given the combination of sonographic findings, β-hCG dynamics, and clinical presentation, laparoscopic exploration was chosen after thorough discussion with the patient and her family, who preferred prompt surgical management.

Preoperative diagnosis of omental pregnancy remains extremely challenging. In our patient, the absence of cervical motion tenderness and adnexal tenderness was atypical for tubal ectopic pregnancy. The omentum is located high in the abdomen and is often obscured by bowel gas, so transvaginal ultrasound has low sensitivity and rarely directly visualizes the lesion (3).

Nevertheless, our case provided key diagnostic clues: an empty uterine cavity, a persistent non-tubal adnexal heterogeneous mass, and progressively increasing pelvic fluid. This constellation of findings is distinctly different from the sonographic picture of typical tubal pregnancy and should prompt clinicians to broaden the differential diagnosis to include abdominal pregnancy. We emphasize that when β-hCG continues to rise and repeated ultrasound fails to locate the pregnancy intrauterine or in the fallopian tube, close communication with the ultrasonographer is essential, and active scanning of the abdominal cavity should be performed.

The negative transabdominal sonographic findings in this case should be interpreted with caution. During laparoscopy, the omental lesion was found to be completely covered by overlying bowel loops, which may explain why transabdominal ultrasonography failed to detect the ectopic mass, as bowel gas frequently creates acoustic shadows that obscure abdominal structures. This observation reinforces the importance of transvaginal ultrasound and a high index of clinical suspicion, rather than reliance on negative abdominal screening.

Although magnetic resonance imaging (MRI) can be valuable for preoperative diagnosis of rare-site ectopic pregnancies (9, 10), MRI requires additional waiting time and cost. Given that the patient and her family preferred prompt surgical management, direct surgical exploration was performed.

### Importance of systematic laparoscopic exploration

When pelvic exploration does not reveal a tubal pregnancy, surgeons may be tempted to stop there, leading to missed diagnosis of an abdominal pregnancy at a rare site. Terrinoni et al. emphasized the importance of systematic exploration to avoid missed diagnosis (11). Lee et al. reported three cases of atypical ectopic pregnancies (including one omental pregnancy) and noted that when pelvic exploration is negative, systematic exploration of the entire abdominal cavity is necessary (12). Minis et al., in a paper specifically summarizing the lessons of laparoscopic management of omental pregnancy, reiterated the extreme importance of systematic exploration, particularly noting that the upper abdomen should not be omitted (13). In our case, after confirming that both tubes, ovaries, and the pelvis were free of gestational tissue, we extended the exploration systematically to the upper abdomen, including the diaphragm, liver surface, bowel, mesentery, paracolic gutters, and remaining peritoneal surfaces. The lesion was eventually found on the omentum, concealed by overlying bowel loops. This systematic approach—from pelvis to upper abdomen, from reproductive organs to peritoneal surface—should be considered when the adnexal findings are negative.

### Surgical considerations

Currently, there is no standardized surgical approach for omental pregnancy, and the extent of resection is based on the surgeon's discretion. In our case, bipolar coagulation was applied to precoagulate the omentum approximately 1 cm around the lesion, followed by complete excision along the precoagulated margin. Postoperatively, serum β-hCG declined to below the upper limit of normal (nonpregnant range) by day 25, and menstruation resumed on day 28, suggesting complete removal of trophoblastic tissue.

In the present case, the left ovarian cyst was removed during the same operation. Intraoperatively, the cyst wall appeared thin, and a small bleeding point was noted on its surface. Given the potential risk of rupture or further bleeding, cystectomy was performed. Histopathology was consistent with a corpus luteum cyst with hemorrhage. The difference between the ultrasound measurement (3.5 cm) and the intraoperative description (5 cm) may reflect the inherent limitations of ultrasound in accurately measuring adnexal lesions. No progesterone supplementation was given after cystectomy, and long-term fertility outcomes will be followed up.

### Risk factors and pathogenesis

The etiology of omental pregnancy is not fully understood. Our patient had no history of pelvic inflammatory disease, abdominal surgery, or assisted reproductive technology. Omental pregnancy has been reported in multiparous women (14), suggesting a heterogeneous background for this condition. Smoking is a well-established risk factor for tubal ectopic pregnancy, but its relationship with primary abdominal pregnancy remains to be studied. In this case, no visible endometriosis was found during laparoscopy. Beyond these risk factors, the underlying mechanisms of trophoblastic migration to rare sites such as the omentum remain to be clarified.

Yuan et al. reported a case of retroperitoneal ectopic pregnancy in which histopathological examination showed that the pregnancy tissue was located inside an enlarged lymph node surrounded by lymphoid tissue, with lymphocyte infiltration also observed in the endometrial tissue. This finding suggested that the implanted embryo may have migrated to the retroperitoneum through lymphatic channels, providing direct pathological evidence for lymphatic migration (15). Hou et al., in a case report and literature review of retroperitoneal ectopic pregnancy, also listed vascular and lymphatic pathways as one of the possible mechanisms by which the fertilized egg may reach the retroperitoneal space, alongside other proposed routes such as post-salpingectomy fistula formation, intestinal compression and peristalsis, and congenital peritoneal defects (16). These mechanisms may represent the pathogenesis of retroperitoneal ectopic pregnancy; however, whether they apply to omental pregnancy remains to be considered.

### Conservative treatment considerations

Methotrexate (MTX) is a commonly used conservative treatment for hemodynamically stable unruptured ectopic pregnancy (1). However, evidence on the efficacy of MTX specifically for omental pregnancy is extremely limited. For example, Koniares et al. reported a case in which β-hCG continued to rise after MTX treatment, eventually requiring surgery (17). In our patient, the pregnancy location was undetermined, the β-hCG level was elevated, and an abdominal pregnancy could not be excluded. After comprehensive discussion with the patient and her family, laparoscopic exploration was chosen rather than conservative treatment.

### Limitations

The limitations of this study include: (1) preoperative MRI was not performed, which may limit the completeness of imaging data; (2) the follow-up period is relatively short, and long-term fertility outcomes require further follow-up; (3) as a single case report, the generalizability of the conclusions is limited.

### Patient perspective

The patient expressed satisfaction with the minimally invasive surgical approach and the rapid postoperative recovery. She reported that her emotional state remained stable throughout the diagnostic and treatment process, and she felt reassured by the close monitoring and clear communication from the medical team.

## Conclusion

Primary omental pregnancy is extremely rare and difficult to diagnose preoperatively. In this case, the omental lesion was concealed by overlying bowel loops and was not identified on preoperative imaging. This case illustrates that when an ectopic pregnancy is strongly suspected but cannot be localized on imaging, systematic laparoscopic exploration extending beyond the pelvis—including careful inspection of the omentum—should be considered to avoid missing a rare implantation site. Such exploration led to the identification and complete excision of the lesion.

## Data availability statement

The original contributions presented in the study are included in the article/Supplementary Material, further inquiries can be directed to the corresponding author.
